# Beyond the face: multidimensional care challenges and unmet needs in Hemifacial Microsomia families

**DOI:** 10.3389/fpubh.2025.1645798

**Published:** 2025-08-07

**Authors:** Yan Huang, Li Gui

**Affiliations:** Faculty of Nursing, Naval Medical University, Shanghai, China

**Keywords:** Hemifacial Microsomia, caregiver burden, unmet medical needs, epidemiology, support group

## Abstract

**Introduction:**

Hemifacial Microsomia (HFM), the second most common congenital facial deformity, significantly impacts patients’ physical appearance and psychosocial well-being, imposing considerable caregiving burdens on families. This study investigates the clinical characteristics of HFM patients, caregiver burdens, and unmet medical needs within Chinese online support communities.

**Methods:**

A cross-sectional study was conducted using convenience sampling of members from an HFM caregiver support group on WeChat APP. Data were collected via electronic questionnaires from March to April 2025, with 141 valid responses. The questionnaire assessed caregiver demographics, the child’s disease characteristics, prenatal history, and surgical experiences. For data analysis, we employed a multifaceted approach, utilizing descriptive statistics to summarize key variables, correlation analysis to explore relationships between factors, and thematic analysis to interpret responses to open-ended questions.

**Results:**

The study included 141 caregivers, mostly females (77.3%) aged 31–50 years (88.65%). Key findings revealed a higher prevalence of HFM in female patients (53.19%) and common comorbidities such as facial cleft (81.6%) and micrognathia (52.5%). Caregivers reported significant financial strain, with monthly household income and educational levels positively correlated with financial burden (*p* < 0.05). Rural residents and unemployed caregivers experienced heavier burdens (*p* < 0.05). Among 95 children with postoperative data, 21.88% expressed dissatisfaction with surgical outcomes, primarily due to unsatisfactory appearance. Additionally, 67.35% of families faced moderate-to-severe care burdens, with 85.11% of caregivers reported heightened sensitivity to social reactions toward their child’s condition.

**Conclusion:**

HFM patients and their families face substantial medical, financial, and psychosocial burdens, including barriers to accessing care, meeting special needs, and receiving health education. Interventions addressing both clinical and emotional support are critical to improving their quality of life. Future research should employ diverse sampling methods and longitudinal studies to enhance the validity of findings on HFM caregiving experiences.

## 1 Introduction

Hemifacial Microsomia (HFM), also known as craniofacial microsomia, the first and second branchial arch syndrome, or lateral facial dysplasia, is the second most common congenital facial deformity with an incidence rate of 1: 3,500–5,600 among live births (1). HFM is characterized by unilateral or bilateral hypoplasia of facial structures, primarily involving the ears, mandible, and other facial components (2). This condition profoundly affects both physical appearance and psychosocial well-being, frequently leading to social stigma, reduced self-esteem, and substantial caregiver burdens (2, 3).

Primary caregivers of children with HFM encounter significant psychological and social challenges, exacerbated by the multidisciplinary medical needs of their children (4). Due to the complexity of HFM, which requires a multidisciplinary surgical approach and long-term rehabilitation, caregivers’ emotional support and financial resources are essential for successful treatment outcomes (5). Thus, understanding caregivers’ experiences and support needs is essential to mitigate the socioeconomic and emotional strains on these families.

Despite expanding treatment options for HFM, many patients lack access to necessary care (6). This may relate to disparities in the distribution of medical resources, the financial status of patients’ families, and limited awareness of HFM. Consequently, investigating the unmet medical needs of HFM patients is urgent to inform targeted interventions.

Although HFM research has grown in recent years, most studies remain limited to single-center analyses or case reports. To overcome this, our study analyzed data from the online patient-support group, gathering diverse patient and family information. Using online platform, we captured real - world experiences and unmet needs of the HFM patients and their families. This study aims to explore the characteristics of HFM patients, the burden impact on families, and the unmet medical needs identified by caregivers within Chinese online community.

## 2 Methods

### 2.1 Participants and procedures

This study used convenience sampling to recruit participants from the WeChat support group for caregivers of children with HFM. Caregivers were eligible to participate if they were primary caregivers of children diagnosed with HFM and were members of the WeChat support group.

Data collection was conducted from March to April 2025 via an electronic questionnaire distributed through WenJuanXing to the WeChat support group. Prior to the formal survey, caregivers provided informed consent. The WeChat support group had a total of 498 members. To enhance participation, the questionnaire was shared several times when group activity was at its peak period. Ultimately, 141 valid questionnaires were collected. The survey had a response rate of 28.3% (141/498), and all returned questionnaires were fully completed. This complete dataset (*n* = 141) was used for the subsequent statistical analysis.

### 2.2 Measures

A self-designed questionnaire was developed to collect data across three domains: Participant Characteristics (caregivers), Patient Clinical Profiles (children), and Maternal Characteristics and Psychological Adaptation (mothers). The questionnaire comprised 46 items and utilized a mixed scoring approach, including categorical options, open-ended questions, and a 5-point Likert scale. Before the formal survey, we pre-tested the questionnaire through expert consultation and a pilot survey with 10 participants to refine the wording and improve clarity.

#### 2.2.1 Caregivers measures

It included 11 items, which captured demographic variables (age, sex, education level, employment status, marital status, urban/rural residence, living city), socioeconomic indicators (monthly household income quintiles, treatment-related financial burden graded as low/moderate/high), support group participation drivers (9 motivation categories), and self-reported caregiving burden severity (5-point Likert scale).

#### 2.2.2 Children measures

It included 20 items, and was divided into two parts, children’s clinical profiles and surgical experience. The clinical profiles including sex, age, birth weight, congenital anomaly subtypes (11 classifications), sensory function status (hearing/vision categorized as normal/abnormal/undetermined), other medical condition, feeding practices (preoperative methods: exclusive breastfeeding, formula feeding, mixed feeding, expressed milk; postoperative adaptations). The surgical experience (cleft repair status, age at surgery, cost, insurance coverage, additional surgery, intervention timing), and treatment experience metrics (satisfaction rate, doctor-patient communication, barrier identification), over all hospital rating and postoperative complaint.

#### 2.2.3 Maternal measures

It included 15 items, which assessed perinatal exposures (maternal age, gestational age, medication use, diabetes/hypertension complications), hereditary/environmental risk factors (familial HFM history, pollution exposure), and psychological adaptation trajectories encompassing post-diagnosis depressive symptoms (asymptomatic/mild/moderate/severe), condition disclosure patterns (non-disclosure/selective disclosure/public disclosure), overprotective parenting frequencies (never/occasional/consistent), and reproductive decision-making among primiparous women, emotional challenges post-diagnosis, behavioral recovery mechanisms.

### 2.3 Data analysis

Data were analyzed using SPSS version 28. Descriptive statistics were employed to summarize demographic and clinical characteristics. Correlation analysis was conducted to examine relationships between core variables and burden in the family. Statistical significance was determined at the *p* < 0.05 level, and effect sizes were reported where applicable. Qualitative data from open-ended questions were thematically analyzed to identify patterns and insights related to caregivers’ experiences and perceptions.

## 3 Results

### 3.1 Participant characteristics

A total of 141 caregivers of HFM patients from an online support group completed the survey. Our survey participants hailed from 31 of China’s 34 provincial administrative regions, indicating a wide and relatively comprehensive data collection scope. This broad representation enhances the generalizability of our findings to the study population to some extent. However, it should be noted that the sample may not be fully representative of all HFM families in China due to the recruitment method through the online support group. The majority of participants were female (77.3%), aged 31–50 years (88.65%), and parents of children with HFM (98.6%). Most of the caregivers had a full-time job (60.99%) or part-time job (22.7%). In addition, Caregivers primarily joined the online support group for medical information acquisition (disease knowledge: 84.4%; treatment guidance: 80.14%) and peer networking (76.6%). Psychological support was sought by 41.13%, while nearly half (43.97%) reported altruistic motivations to assist fellow patients. Detailed demographic data are presented in Table 1.

**Table 1:** Caregiver sociodemographic characteristics (*n* = 141).

Caregiver profile	*n*	[%]
Sex		
Male	32	22.7%
Female	109	77.3%
Age group (years)		
18–30	16	11.35%
31–50	125	88.65%
>50	0	0%
Education		
Middle school	13	9.22%
High school	64	45.39%
Bachelor degree	57	40.43%
Master or PHD	7	4.96%
Employment status		
Full-time	86	60.99%
Part-time	32	22.7%
Unemployed	22	15.6%
Maternity leave	1	0.71%
Marital status		
Married	134	95.04%
Single/ divorce / bereave	7	4.96%
Residence		
Urban area	104	73.76%
Rural area	37	26.24%
Monthly household income (¥)		
<5,000	25	17.73%
5,001–10,000	59	41.84%
10,001–20,000	28	19.86%
20,001–30,000	20	14.18%
>30,001	9	6.38%
Financial burden related to the treatment		
High strain	47	33.33%
General strain	83	58.87%
No strain	11	7.8%
Self-rated caregiver burden		
None	17	12.06%
Mild	22	15.6%
Moderate	70	49.65%
Severe	26	18.44%
Extremely severe	6	4.26%
The reason of join the support group online		
Acquiring disease knowledge or scientific research progress	119	84.4%
Seeking psychological support (to alleviate negative emotions)	58	41.13%
Seeking hospital, medication, or nursing guidance	113	80.14%
Strengthening patient relationships and exchanging medical information	108	76.6%
Daily conversations enrich oneself and find a sense of belonging.	18	12.77%
Hope to learn some parenting experience	37	26.24%
Hope to help some patients in need	62	43.97%
Purchase or transfer medical resources (drugs, equipment, etc.)	5	3.55%
Other	3	2.13%

The average household in China earned 12,180 yuan per month in Q1 2025 (based on a standard three-person household) (7). In our study, more than 60% of the households earned below the average household. The Kendall’s coefficient was used to analysis the correlation between various factors and the treatment-related financial burden on the family among 141 participants (Table 2). Monthly household income had a significant positive correlation with financial burden (*r* = 0.472, *p* < 0.01). Job status (*r* = −0.227, *p* < 0.01) and resident area (*r* = −0.194, *p* < 0.05) showed significant negative correlations. Education level also had a significant positive correlation (*r* = 0.308, *p* < 0.01). The results indicate that higher income and education levels are associated with lower financial burden, while unemployment and rural residency are linked to higher burden. Overall, the financial strain correlated strongly with caregiver burden levels, where 72.35% self-reported moderate-to-extremely severe burden. The highest prevalence occurred in the moderate burden category (49.65%), indicating substantial care-related impacts.

**Table 2:** Kendall’s correlation of treatment-related financial burden.

Variable	Treatment-related financial burden
Monthly household income (¥)	0.472**
Job status	−0.227**
Education level	0.308**
Residence (Urban/Rural)	−0.194*

* *p* < 0.05 ** *p* < 0.01. Values represent Kendall’s correlation coefficients, indicating the strength and direction of association between perceived treatment cost burden and demographic variables. Coefficients range from −1 (perfect negative) to +1 (perfect positive), with 0 indicating no association. Key findings: higher income and education levels are associated with lower financial burden, while unemployment and rural residency are linked to higher financial burden.

### 3.2 Patient clinical profiles

#### 3.2.1 Baseline characteristics of the children

Among 141 children with HFM, 66 (46.81%) were boys and 75 (53.19%) were girls. The mean age at enrollment was 45.45 ± 36.42 months, and the average birth weight was 3.27 ± 0.51 kg. Before surgery, feeding methods varied: 49 (34.75%) were breastfed, 11 (7.8%) were bottle-fed with breast milk, 47 (33.33%) had mixed feeding (breastfeeding and bottle-fed formula), and 34 (24.11%) were bottle-fed with both breast milk and formula. Of these, 95 (67.38%) underwent transverse cleft repair. Postoperatively, most maintained their preoperative feeding method (breastfeeding: 44.79%; bottle-feeding: 40.63%), while only 5.21% transitioned from bottle to direct breastfeeding (Table 3).

**Table 3:** Baseline characteristics of HFM children (*n* = 141).

Children profile	*n* [%] or Mean ± SD	
Sex		
Boy	66	46.81%
Girl	75	53.19%
Current age (months)	45.45 ± 36.42	
Birth weight (kg)	3.27 ± 0.51	
Congenital anomalies (multiple choice)		
Facial cleft		
Left lateral	45	31.91%
Right lateral	65	46.1%
Bilateral	5	3.55%
Ear tag		
Ipsilateral	44	31.21%
Bilateral	25	17.73%
Facial tag		
Ipsilateral	25	17.73%
Bilateral	4	2.84%
Tragus absence		
Ipsilateral	15	10.64%
Bilateral	2	1.42%
Microtia		
Unilateral	52	36.88%
Bilateral microtia	1	0.71%
Micrognathia		
Mild	24	17.02%
Severe	50	35.46%
Preauricular fistula	17	12.06%
Absence of auricle	6	4.26%
External auditory canal cleft	0	0%
External auditory canal atresia	30	21.28%
Other	13	9.22%
Hearing function		
Normal	95	67.38%
Abnormal	35	24.82%
Unknown	11	7.8%
Visual function		
Normal	117	82.98%
Abnormal	20	14.18%
Unknown	4	2.84%
Other medical conditions		
No	105	74.47%
Yes	36	25.53%
Feeding method pre-surgery		
Exclusive breastfeeding	49	34.75%
Bottle (breast milk)	11	7.8%
Mixed feeding	47	33.33%
Bottle (formula)	34	24.11%
Undergone the transverse cleft repair		
No	46	32.62%
Yes	95	67.38%
Has the feeding method changed after surgery? (*N* = 95)		
No change, always breastfeeding	43	44.79%
No change, always bottle-feeding	39	40.63%
Changed, switched from bottle-feeding to direct breastfeeding	5	5.21%
Other method	9	9.38%

Multi-site malformation involvement was common in the children with HFM. The lateral facial cleft predominated, with right-sided involvement (46.1%) exceeding left-sided (31.91%) and bilateral presentations (3.55%). Microtia affected 36.88% of cases (unilateral: 36.88%, bilateral: 0.71%), while mandibular hypoplasia was observed in 52.48% (mild: 17.02%, severe: 35.46%). Auditory anomalies included external canal atresia (21.28%) and preauricular fistulae (12.06%). Among these cases, 48.94% displayed ipsilateral or bilateral ear tags, and 20.57% exhibited facial tags. Moreover, regarding the sensory functional impairments, 35 (24.82%) cases exhibited hearing deficits, and 20 (14.18%) cases had visual abnormalities. Notably, 10 children (7.09%, 6 boys and 4 girls) had both hearing and vision impairments. There were 36 (25.53%) cases reported other medical conditions, such as dermoid tumor, congenital heart disease, patent foramen ovale, scoliosis, polydactyly, craniosynostosis, unilateral renal agenesis, etc.

#### 3.2.2 Postoperative outcomes of the children

**Figure 1:**
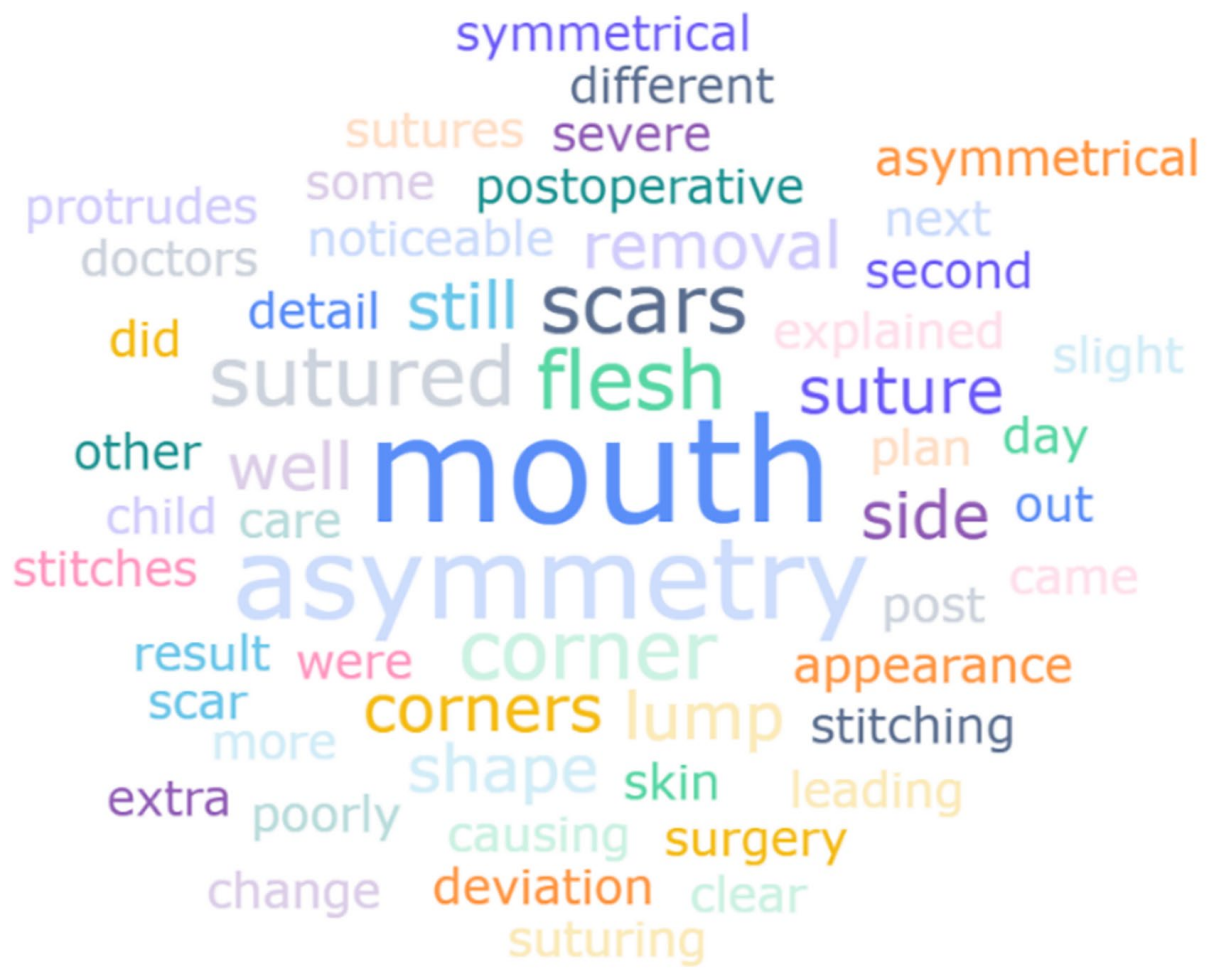
Word cloud of postoperative complaint themes.

Among 95 children who underwent transverse cleft repair, the mean age at surgery was 9.14 ± 8.43 months (Table 4). Additional surgeries were required in 31.25% of cases. Parental satisfaction with surgical outcomes revealed varied perspectives. There were 35.42% of parents expressed satisfaction, 42.71% remained neutral, and 21.88% reported dissatisfaction. Postoperative complaints were visually summarized in Figure 1 through a word cloud analysis, which highlights the frequency of specific concerns based on caregiver-reported qualitative responses. The most prominent terms included “mouth,” “asymmetry,” “scars and “suture,” reflecting dissatisfaction with esthetic outcomes. For instance, specific complaints included “poor shape of mouth corner sutures,” “misaligned corners of the mouth,” and “long scar.”

**Table 4:** Surgical experiences and barriers (*n* = 95).

Child’s profile	*N* [%] or Mean (SD)	
Age at surgery (months)	9.14 ± 8.43	
Undergone additional surgeries		
No	66	68.75%
Yes	30	31.25%
Hospitalization cost for the transverse cleft repair (¥)	15,042 ± 10,749	
Insurance coverage		
No	45	46.88%
Yes	51	53.13%
Satisfaction with surgery outcome		
Satisfied	34	35.42%
Neutral	41	42.71%
Dissatisfied	21	21.88%
Were you informed by your doctor about how your baby’s surgery would be conducted?		
No	24	25%
Yes	72	75%
Were you told about what technique used for suturing?		
No	48	50%
Straight line method	19	19.79%
Z-plasty	24	25%
W-plasty	4	4.17%
Dual triangular flap method	1	1.04%
Rectangular flap method	0	0%
Would you recommend this doctor to other patients?		
Yes	59	61.46%
No	37	38.54%
Barriers during treatment (multiple choice):		
No significant barriers	34	35.42%
Medical facility is too far away	44	45.83%
Treatment cost is too high	18	18.75%
Unsure which doctor/department to consult	22	22.92%
Difficulty in making an appointment	23	23.96%
Lack of postoperative care knowledge	30	31.25%
Other [detailed]	8	8.33%
Overall hospital rating		
Very satisfied	19	19.79%
Satisfied	42	43.75%
Neutral	32	33.33%
Poor dissatisfied	2	2.08%
Very dissatisfied	1	1.04%

Despite the relatively low satisfaction rate, 61.46% of parents would recommend their surgeon to other patients. Notably, preoperative communication gaps were evident, as 25% of parents received no explanations regarding surgery, and 50% were unaware of the suture methods used. Furthermore, the three most prevalent treatment barriers were identified as geographic inaccessibility (45.83%), followed by insufficient postoperative care education (31.25%), and systemic challenges in appointment scheduling (23.96%). Postoperative concerns of the caregivers were focused on the development of the child, such as “facial asymmetry,” “hearing problems,” “future treatment plan,” “child’s psychological problems,” etc.

### 3.3 Maternal characteristics and psychological adaptation

#### 3.3.1 Perinatal characteristics and maternal demographics

The retrospective cohort comprised 141 mothers with a mean age of 29.58 ± 4.46 years during pregnancy. Most pregnancies reached full term (90.07%), with preterm and post-term births accounting for 7.09 and 2.84%, respectively. Medication use during pregnancy was reported by 75.89% of participants, while gestational diabetes (13.48%) and hypertension (3.55%) constituted the primary complications. There were 3.55% of mothers reported family history of HFM, and 4.96% reported clearly identified environment pollution during pregnancy (Table 5).

**Table 5:** Perinatal characteristics and maternal demographics (*n* = 141).

Maternal profile	*N* [%] or Mean (SD)	
Maternal age at pregnancy (years)	29.58 ± 4.46	
Gestational age at birth		
Preterm (<37 wks.)	10	7.09%
Full-term (37–42 wks.)	127	90.07%
Post-term (>42 wks.)	4	2.84%
Prenatal medication use		
Yes	107	75.89%
No	34	24.11%
Pregnancy complications		
Gestational diabetes mellitus	19	13.48%
Gestational hypertension	5	3.55%
No	118	83.69%
Family history of HFM		
No	136	96.45%
Yes	5	3.55%
Clearly identify pollution factors in the work environment		
No	134	95.04%
Yes	7	4.96%

#### 3.3.2 Postnatal psychological and behavioral adaptation

**Figure 2:**
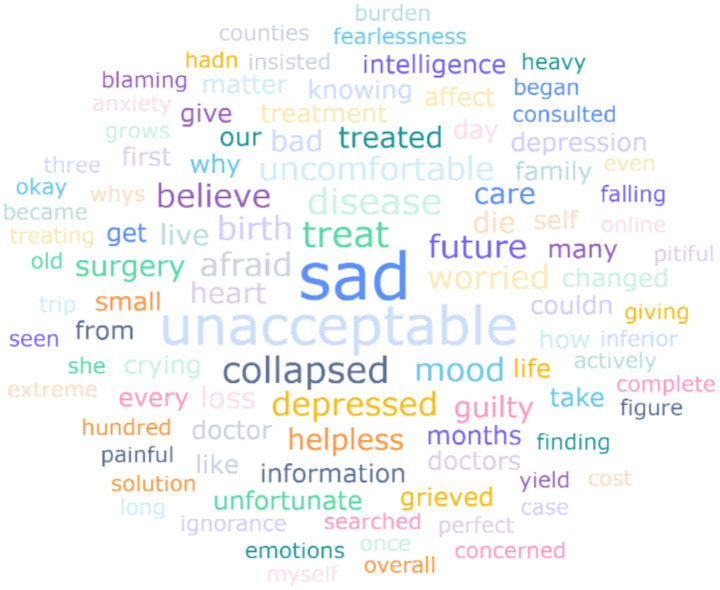
Word cloud of emotional changes post-diagnosis.

The HFM condition was prenatally detected in only 4.97% of cases (2.84% via 2D ultrasound; 2.13% via 3D/4D imaging). Behavioral analysis showed 47.52% of mothers exhibited some degree of overprotection, with 8.51% demonstrating frequent overprotective behaviors. Social awareness concerns were prevalent, with 85.11% expressing sensitivity to others’ reactions toward their child’s condition. Among first-time parents (*n* = 86, 61.00%), 39.53% expressed no intention to have additional children, while 31.40% reported hesitancy due to concerns about disease recurrence in future offspring (Table 6). Following diagnosis disclosure, most parents experienced negative emotions. Figure 2 presents a word cloud derived from open-ended responses describing caregivers’ emotional states post-diagnosis. Dominant terms like “sadness,” “unacceptable,” and “treatment” dominated the visual field, with smaller but recurrent terms such as “depress,” “guilty” and “uncomfortable” revealing nuanced psychological impacts. This aligns with the 68.1% of mothers reporting depressive symptoms (Table 6), suggesting that emotional distress is both prevalent and multifaceted. Specifically, 50.35% of mothers developed mild depression, 14.18% progressed to moderate depression, and 3.55% developed severe depressive states. Among those experiencing depression (*n* = 96), 52.08% reported symptom alleviation through personal coping strategies, while 17.71% benefited from the online support group.

**Table 6:** Postnatal psychological and behavioral adaptation (*n* = 141).

Maternal profile	*n* [%]	
Prenatal HFM or transverse cleft detection		
No	134	95.04%
Yes, detected through 2D ultrasound	4	2.84%
Yes, detected through 3D/4D ultrasound	3	2.13%
Condition disclosure to others after diagnosis		
No, no one informed	11	7.8%
Yes, only some very close friends and family members were informed	109	77.3%
Yes, I do not care. I’ve informed most of them	21	14.89%
Being sensitive to others’ reactions to children, due to the condition		
Very concerned about others’ attitudes	29	20.57%
A little concerned about others’ attitudes	91	64.54%
It does not matter about others’ attitudes	21	14.89%
Overprotective behaviors due to the disease		
Frequent	12	8.51%
Occasional	67	47.52%
None	62	43.97%
Birth order		
First child	86	61.00%
Second child	52	36.88%
Third child	3	2.13%
Future reproduction plans if it’s your first child?		
No	34	39.53%
Plan more children	25	29.07%
Hesitant due to recurrence risk	27	31.4%
Do you have a tendency toward depression after learning about your baby’s condition?		
No depression	45	31.91%
Mild depression	71	50.35%
Moderate depression	20	14.18%
Severe depression	5	3.55%
Has your depression recovered after surgery? (*N* = 96)		
No change, I still often feel depressed	11	11.46%
Accepting the reality through personal insight has alleviated my depressive symptoms	50	52.08%
After the baby’s surgery, I was engaged intensely in infant care which prevented the onset of depressive symptoms.	11	11.46%
After being comforted by friends and family, things gradually improved	7	7.29%
Improved after joined the patient online support group	17	17.71%

## 4 Discussion

HFM, the second most common craniofacial malformation after cleft lip and palate, is marked by one-side face underdevelopment, impacting jaw, ears, and zygomatic arch (8). It causes lifelong economic burdens on families and poses multisystem challenges for patients. To enhance the quality of life and inform healthcare policies, understanding HFM’s epidemiology, caregiving burdens, and unmet needs is crucial. Our research addresses this by using online support groups to collect patient-reported data, analyzing HFM’s epidemiology, caregiving challenges, and unmet medical needs from the patients’ perspective.

### 4.1 Epidemiological characteristics of HFM children

#### 4.1.1 Gender difference

The gender distribution of HFM has been a subject of interest and debate in the literature. While most studies report more male cases (9, 10), our study found a higher prevalence among females (53.19%), with a female-to-male ratio of 1.4:1. This finding contrasted with the majority of existing literature but aligns a smaller subset of studies, such as a study conducted in Egypt (2), the USA (11). Moreover, it has been reported that there was an absence of significant gender predominance in cases of HFM (12). The inconsistencies may arise from regional variations, cultural factors, or biological differences influencing gender-specific HFM susceptibility. Families with female HFM patients are more likely to be aware of minor facial asymmetries, which may prompt them to actively participate in various activities and seek treatment methods. In contrast, males may underreport such symptoms. However, it should be noted that the gender distribution in our study may stem from selection bias due to the web-based survey and online recruitment method, so the results might not reflect national trends and should be interpreted cautiously. Future research should probe into these factors to clarify the mechanisms behind HFM’s gender differences.

#### 4.1.2 Comorbidity profile

HFM patients often present with multiple comorbidities of varying severity, leading to diverse clinical manifestations and impacting their quality of life and treatment outcomes. Our study found a high incidence of comorbidities in HFM patients, such as facial cleft (81.6%), micrognathia (52.5%), ear deformities (49.6%), ear tag (48.9%), facial tag (20.6%). Unilateral onset was seen in about 95% of patients, with the right side more often affected than the left, in line with other literature results (6, 13). Heart defects (0.05%), dermoid tumor (0.06%) and spinal deformities (0.02%) were also reported. Furthermore, these defects not only affect appearance but also cause functional problems like chewing, suctioning, pronunciation, and hearing. Our study revealed a high burden of sensory deficits: hearing impairment affected 24.8% of cases, visual abnormalities 14.2%, and concomitant audiovisual impairment 7.1% (*n* = 10; M:F = 1.5:1). It should be noted that these prevalence rates likely underestimate the true burden due to the caregiver-reported methodology, where unilateral hearing loss may evade detection through contralateral compensation. Future studies should incorporate objective hearing tests to accurately detect hearing loss in children with HFM and thereby enable early diagnosis and intervention, which can significantly enhance management outcomes for affected individuals.

#### 4.1.3 Feeding challenges

Infant feeding practices play a pivotal role in shaping both physical growth and psychosocial well-being in infants with HFM and the mother. Our findings revealed that 65.25% of infants with HFM received bottle-feeding or mixed feeding (breast milk and formula), a proportion significantly exceeding rates reported in healthy infant populations (14). Studies have shown that bottle feeding can lead to physiological limitations such as low masseter muscle activity and insufficient sucking efficiency in infants, which may compromise nutrient intake efficiency and oral motor developments (15). Conversely, breastfeeding has been linked to enhanced mother-infant bonding, a protective factor against postpartum depression (16). However, infants with HFM often face structural challenges (lateral or both-side facial cleft) that physically impede effective breastfeeding, forcing caregivers to rely on alternative feeding methods. This feeding limitation may exacerbate maternal psychological distress, as mothers reporting difficulties in breastfeeding exhibited heightened depressive symptoms. Therefore, it is possible that the mother has a higher tendency toward depression. These findings underscore a critical interplay between feeding barriers and maternal mental health in HFM populations. Future studies should explore targeted interventions to support breastfeeding feasibility while addressing caregiver mental health needs to mitigate this dual burden.

#### 4.1.4 Etiology of HFM

The etiology of HFM is not fully understood, but increasing research suggests that it may be related to abnormal migration of neural crest cells, vascular abnormalities or hemorrhage, genetic factors, external environmental factors, and maternal factors (3, 8, 17, 18). Our study revealed elevated maternal gestational diabetes mellitus (GDM) prevalence (13.48%) in HFM cohorts compared to the general pregnancy rates (6.5–9.4%). These findings align with prior studies demonstrating that maternal metabolic dysfunction may impair craniofacial development through disrupted nutrient delivery and vascular signaling (3, 19). Additionally, 3.55% cases of gestational hypertension were reported, which may link to more severe placental hemodynamic abnormalities (19, 20). Notably, 75.89% of mothers reported prenatal medication use, raising concerns about iatrogenic contributions, though specific agents remained unspecified in our study (3, 21). Workplace environmental exposure was reported at 4.96%, which is concerning as emerging evidence links air pollutants and endocrine disruptors to branchial arch developmental anomalies (22). Family history rates (3.55%) fell below genetic studies reporting 6% pathogenic mutations, suggesting under ascertainment of mild familial cases or polygenic inheritance patterns. Overall, our findings underscore the multifactorial nature of HFM etiology, where maternal factors, environmental exposures, and genetic predispositions may interact during critical developmental periods to influence craniofacial morphogenesis. Future research with prospective designs and detailed exposure assessments is needed to further clarify these complex relationships and identify specific risk factors.

### 4.2 Caregiver demographics and burden dynamics

#### 4.2.1 Gender disparities in caregiving

The caregiving job was highly stressful, especially to the family facing the new born baby with unexpected disease (23, 24). The burden of caregiving for children with HFM disproportionately impacts maternal health and family cohesion, with sociocultural norms exacerbating gender disparities in care responsibilities. Our study revealed that 77.3% of primary caregivers were female, with 83.69% actively employed—a marked disparity compared to U.S. cohorts where 60% of caregivers are women and 75% employed in general (25). This imbalance reflects entrenched cultural expectations in China, where maternal responsibility for pediatric care remains normative. Employed mothers experienced multiple pressures, including caring for their baby, managing workplace responsibilities, and coordinating medical treatments. These demands often disrupted household routines and put strain on marital relationships. Such gender - based family division of labor puts a heavier burden on women, affecting not only the caregivers themselves but also the overall family dynamics and disease management process. These findings call for multifaceted interventions, including employer policies supporting flexible work hours and government-funded programs, to redistribute caregiving burdens and mitigate gender-specific vulnerabilities in HFM-affected family.

#### 4.2.2 Economic burden on caregivers

The economic burden impacts caregivers of rare disease patients through direct expenses, indirect economic impacts, psychological distress, and diminished quality of life (26). In our study, 33.33% of caregivers reported high financial strain, a proportion similar to that among cancer patient caregivers (27). Conversely, only 7.8% of caregivers indicated no financial strain. The financial strain experienced by caregivers is closely associated with their employment status and education level. Notably, income and education level were positively correlated with economic burden (*p* < 0.05), while rural residents experienced heavier burdens (*r* = −0.194, *p* < 0.05). These trends align with broader socio-economic patterns in the general population. However, HFM families face unique challenges due to the specialized care their children require, such as multiple surgeries, long-term follow-up, and multidisciplinary interventions for comorbidities like facial cleft and micrognathia (28). This specialized care is more complex and costly than routine healthcare, resulting in a more pronounced financial burden compared to families of children without disabilities. Additionally, 21.88% of caregivers reported dissatisfaction with surgical outcomes, primarily due to appearance concerns, and 67.35% of families experienced moderate-to-severe caregiving burdens, with 85.11% of caregivers being sensitive to social reactions. Unlike the general population, the economic burden in HFM families is more strongly influenced by the child’s specific medical needs and their access to specialized care. Access to specialized healthcare is particularly challenging for rural and unemployed caregivers. These individuals often encounter additional barriers, such as limited medical service availability, transportation difficulties, and inadequate insurance coverage, which further intensify their financial burden. These findings underscore the distinctive economic challenges encountered by HFM families, suggesting that while some socio-economic factors may be universal, the intensity and complexity of their economic burden are uniquely pronounced. Future research should explore these differences by comparing the financial and caregiving experiences of HFM families with those of families with children without disabilities or other chronic conditions.

#### 4.2.3 Mental health status of caregivers

The mental health status of caregivers is a crucial factor affecting the quality of patient care and the psychosocial burden on families. Our study found that the depression rate among mothers of children with HFM was higher than that reported in some studies on mothers of children without disabilities. A meta-analysis of 15,586 women showed that the comorbidity rate of all types of depression within 1 year postpartum was 12.1%, with severe depression accounting for 7.0% (29). Our study revealed that 68.1% of mothers caring for children with HFM reported mild to severe depression, with recurrent negative themes of “cannot accept” and “very sad” dominating their post-diagnosis experiences. These results align with prior evidence indicating that caregivers of children with rare diseases face profound psychological strain, with up to 90% experiencing anxiety, depression, or stress (30). However, our slightly lower depression rates may reflect cultural resilience or methodological differences, while the dual burden of managing complex medical needs—frequent hospital visits, surgeries—while suppressing personal grief left mothers trapped in chronic emotional exhaustion. Caregivers, especially the mother of the HFM child, not only need to manage the psychological and physical needs of the child but also grapple with their own emotional issues, leading to a significant decline in their mental health. Furthermore, the psychological burden of caregivers is closely related to the severity of the patients’ condition, the duration of caregiving, and the lack of social support (31). There are research suggested that a multi-level social support network that combines formal and informal support systems was needed to address issues of reducing caregiver burden, enhancing family resilience, and optimizing long-term disease management through policy and cultural adaptation (32). Thus, future research and policy initiatives should focus on developing comprehensive support strategies to address these challenges and improve outcomes for both caregivers and patients.

### 4.3 Social discrimination and online support group

#### 4.3.1 Limited social support networks

Social stigma and lack of support significantly impact how families of children with HFM disclose their child’s condition. Many caregivers have reported that the lack of an effective social support system makes them feel isolated and helpless during the caregiving process. Our study found that only 14.89% of families opted to disclose their child’s illness publicly, and 77.3% of families decided to share the information only with a small circle of individuals. Notably, 7.8% of families preferred to keep the matter entirely confidential. These findings highlight the diverse approaches families take regarding the disclosure of their child’s health condition. Social stigma related to the child and family with rare disease also played an important role in caregivers regarding their caring behavior. In our study, 85.1% of caregivers were sensitive to others’ reactions to children, due to the illness, and about 56% of caregivers has self-reported overprotection behavior. Studies showed that caregivers’ disclosure decisions regarding children’s illness were shaped by multifaceted social and psychological factors, including disclosure motivations, types of stigma, cultural context, and intergenerational differences, highlighting the need for interventions that both reduce stigma and enhance disclosure capabilities (33, 34). Therefore, by equipping caregivers to reclaim their narratives—turning shame into solidarity—we can break the stigma-isolation cycle and build support networks rooted in empathy, not erasure.

#### 4.3.2 Role of online support group

The reliance on online support group by families affected by HFM underscores the systemic gaps in rare disease care. With limited physician expertise and public awareness, majority of the families in the WeChat online support group primarily joined to gain disease knowledge (84.4%), seek medical guidance (80.14%), and build patient relationships (76.6%). These results highlight the crucial role of online support group in addressing the unique challenges of rare conditions like HFM, where limited medical expertise and public awareness often lead to inadequate social support. Online platforms serve as vital tools for information sharing, emotional support, and community building (35, 36), despite potential issues such as information inaccuracy and lack of coordination with healthcare systems (37). To ensure the effectiveness and safety of these groups, robust technology and appropriate policies are needed to regulate and integrate them into the broader healthcare landscape. This would help create a more comprehensive support system that combines both online and offline resources.

### 4.4 Unmet medical needs

#### 4.4.1 Barriers to healthcare access

The rare disease field is marked by diagnostic delays and resource shortages (38). Families of children with HFM encounter multifaceted barriers to healthcare access, extending beyond financial constraints to encompass geographic, social, and systemic factors. Previous study highlights that numerous patients face challenges in accessing timely medical services, primarily due to limited medical resources, the uneven distribution of healthcare facilities, and inadequate awareness of specific diseases (39). Patients with HFM often require multidisciplinary comprehensive treatment, including plastic surgery, otolaryngology, and dentistry, but due to the concentration of these specialized medical services in large cities, rural families experience significant challenges in accessing specialized care (40). In our study, 24.21% of families were from rural areas, and these families demonstrated a 7.07% lower likelihood of undergoing subsequent facial repair procedures compared to urban families (*p* = 0.43). Though not statistically significant, this trend suggests a potential disparity in healthcare access and utilization between rural and urban settings that warrants further investigation. In addition, patients often encounter the complexity and opacity of the healthcare system when seeking medical assistance, which makes them feel confused and helpless when choosing appropriate medical services. In some cases, the socio-economic status of patients can also affect their ability to access medical care. Research has shown that low-income patients are more likely to face the burden of medical expenses, leading to delayed or abandoned treatment (41).

#### 4.4.2 Unmet special care needs

Caregivers generally report that the quality of healthcare service directly impacts their mental health (42). Due to the age variety in the study group, we only investigated the surgical satisfaction rate related to the transverse cleft repair. We identified multiple unmet medical needs among children who had undergone transverse cleft repair. Transverse cleft repair is the first surgery that infants with HFM may undergo, typically performed after the infant is 3 months old and has reached an appropriate weight. In our study, 95 children (67%) underwent transverse cleft repair, the mean age at surgery was 9.14 ± 8.43 months, which was in the recommended surgical age range of 3 to 12 months (43). However, nearly one-fifth (21.88%) of the family indicated dissatisfaction with the surgery, primarily due to esthetic concerns such as commissural migration, noticeable asymmetry, and lengthy scars. The word cloud analysis (Figure 1) reveals a persistent disconnect between functional and esthetic surgical outcomes, with frequent terms like “asymmetry” and “scar” highlighting unmet cosmetic expectations. This underscores the need for surgical protocols that equally prioritize anatomical correction and esthetic refinement, coupled with improved preoperative counseling to align expectations. However, there is a lack of data to compare the satisfaction rate of the surgery, and the long-term risk of increased scarring and postoperative angular displacement.

#### 4.4.3 Doctor-patient communication

In terms of doctor-patient communication, our study revealed that 25% of parents did not receive necessary preoperative explanations and 50% were unaware of the specific surgical methods or techniques, indicate systematic flaws in the current medical information process. Such deficiencies may foster unrealistic parental expectations regarding surgical outcomes, attributable to insufficient comprehension of the procedure. This underscores the need for more balanced and comprehensive communication strategies in clinical practice. Informed consent goes far beyond the act of signing a paper. Beyond explaining surgical necessity, doctors should prioritize comprehensive informed consent discussions, explicitly detailing operative approaches, potential complications, and realistic outcomes (44).

#### 4.4.4 Health education

Health education plays an important role in the medical management of patients with HFM, but the current resources for health education are still insufficient. Many patients and their families have limited knowledge about HFM, which directly affects their choice and compliance with treatment plans (39). For instance, 31.25% of families in our study reported inadequate postoperative care education. Patients who undergo transverse cleft repair and lack sufficient knowledge may experience suboptimal outcomes. Additionally, there may be a connection between the need for secondary surgeries and the quality of postoperative care. These findings indicate a need for improvements in the standardization of discharge guidance and its implementation.

## 5 Limitations and future directions

The present study provides important insights into the prevalence and caregiving experiences associated with HFM. However, several limitations must be acknowledged that may affect the generalizability and reliability of our findings. Firstly, our online recruitment strategy may overrepresent caregivers with higher health literacy or distress levels, which may have introduced selection bias, limiting generalizability to HFM families outside support groups. The use of online questionnaires may have further excluded families in rural regions with limited internet access, narrowing the sample’s diversity. Moreover, the absence of clinical validation for the data means that self-reported information might be subject to bias, which could influence the reliability of the results. The relatively small sample size and the low response rate of 28.3% also suggest that our sample may not fully capture the complexity of the HFM caregiving experience or represent the broader population affected by HFM. Broader population-level estimates of prevalence and caregiving burdens may therefore differ significantly from our findings, and our results should be interpreted with caution, particularly when extrapolating to populations outside the scope of our study. Future research should employ more diverse and representative sampling strategies, such as combining online and offline recruitment methods and incorporating data from various regions and healthcare settings. This approach may enhance the representativeness of the sample and provide a more comprehensive and accurate estimation of HFM prevalence and caregiving experiences. Longitudinal studies could also offer valuable insights into the incidence, progression, and long-term impacts of caregiver burdens, as well as the effectiveness of support systems over time, thereby enriching the understanding of the HFM caregiving experience. Additionally, validating data through clinical assessments would strengthen the reliability of reported information.

## 6 Conclusion

This study highlights the significant burdens faced by HFM patients and their families. HFM creates challenges in medical care access, special care needs, and health education. Our findings underscore the heavy burden on caregivers and the necessity for comprehensive support programs that address both the medical and emotional needs of children with HFM and their caregivers. We suggest that improving medical resource allocation, enhancing nursing professionalism, and providing effective health education could greatly enhance patients’ quality of life and treatment outcomes. Furthermore, we recommend the establishment of standardized HFM care pathways that incorporate multidisciplinary teams and mandatory mental health screening for caregivers. Future research should prioritize longitudinal evaluations of surgical outcomes and caregiver support models, with a focus on their applicability across different socioeconomic settings in China.

## Data Availability

The raw data supporting the conclusions of this article will be made available by the authors, without undue reservation.

## References

[1] Young A Spinner A. Hemifacial Microsomia. Treasure Island, FL: StatPearls Publishing (2023). 32809654

[2] Allam KA. Hemifacial microsomia: clinical features and associated anomalies. J Craniofac Surg. (2021) 32:1483–6. doi: 10.1097/SCS.0000000000007408, PMID: 33587521

[3] Luo S Sun H Bian Q Liu Z Wang X. The etiology, clinical features, and treatment options of hemifacial microsomia. Oral Dis. (2023) 29:2449–62. doi: 10.1111/odi.14508, PMID: 36648381

[4] Bini A Derka S Stavrianos S. Hemifacial microsomia surgical approach and anotia reconstruction: a case report. In Vivo. (2024) 38:2550–6. doi: 10.21873/invivo.13729, PMID: 39187366 PMC11363786

[5] Pattnaik A Lim A Sabeti S Kwon A Hall K Lott I. A unique case of progressive hemifacial microsomia or parry-romberg syndrome associated with limb and brain anomalies with normal neurological findings: a review of the literature. Eur J Med Genet. (2021) 64:104234. doi: 10.1016/j.ejmg.2021.104234, PMID: 34082156

[6] Jia Y Zhang Z Gao S Jiang F Wang Y Su X. Evaluation of situs inversus combined with plastic surgery–related malformations in a Chinese clinic population. Ann Plast Surg. (2025) 94:217–21. doi: 10.1097/SAP.0000000000004159, PMID: 39652841

[7] National Bureau of Statistics. (2025). Resident income and consumption expenditure in the first quarter of 2025. Available online at: https://www.stats.gov.cn/xxgk/sjfb/zxfb2020/202504/t20250416_1959322.html [Accessed July 5, 2025]

[8] Paul MA Opyrchał J Knakiewicz M Jaremków P Bajtek J Chrapusta A. Hemifacial microsomia review: recent advancements in understanding the disease. J Craniofac Surg. (2020) 31:2123–7. doi: 10.1097/SCS.0000000000006616, PMID: 33136839

[9] Caron CJJM Pluijmers BI Wolvius EB Looman CWN Bulstrode N Evans RD. Craniofacial and extracraniofacial anomalies in craniofacial microsomia: a multicenter study of 755 patients. J Craniofac Surg. (2017) 45:1302–10. doi: 10.1016/j.jcms.2017.06.001, PMID: 28684073

[10] Kim BS Chen X Chen C Chong CH Yan YJ Han W. OMENS+ classification correlations analysis of craniofacial microsomia in China: the relationship between macrostomia and mandibular hypoplasia. J Craniofac Surg. (2022) 33:1126–9. doi: 10.1097/SCS.0000000000008247, PMID: 35045015

[11] Vento AR LaBrie RA Mulliken JB. The O.M.E.N.S. Classification of hemifacial microsomia. Cleft Palate Craniofac J. (1991) 28:68–76. doi: 10.1597/1545-1569_1991_028_0068_tomens_2.3.co_2 1848447

[12] Xu S Zhang Z Tang X Yin L Liu W Shi L. The influence of gender and laterality on the incidence of hemifacial microsomia. J Craniofac Surg. (2015) 26:384–7. doi: 10.1097/SCS.0000000000001336, PMID: 25723655

[13] Zhang Z Li X Chen X Sun M Kim BS Aung ZM. Correlations between mandible condylar structures and external ear deformities in hemifacial microsomia with three-dimensional analysis. J Craniofac Surg. (2022) 33:1154–8. doi: 10.1097/SCS.0000000000008342, PMID: 34743153

[14] Kotowski J Fowler C Hourigan C Orr F. Bottle-feeding an infant feeding modality: an integrative literature review. Matern Child Nutr. (2020) 16:e12939. doi: 10.1111/mcn.12939, PMID: 31908144 PMC7083444

[15] França EC Sousa CB Aragão LC Costa LR. Electromyographic analysis of masseter muscle in newborns during suction in breast, bottle or cup feeding. BMC Pregnancy Childbirth. (2014) 14:154. doi: 10.1186/1471-2393-14-154, PMID: 24885762 PMC4014087

[16] Westerfield KL Koenig K Oh R. Breastfeeding: common questions and answers. Am Fam Physician. (2018) 98:368–73. PMID: 30215910

[17] Wang Y Ping L Luan X Chen Y Fan X Li L. A mutation in VWA1, encoding von willebrand factor a domain-containing protein 1, is associated with hemifacial microsomia. Front Cell Dev Biol. (2020) 8:571004. doi: 10.3389/fcell.2020.571004, PMID: 33015062 PMC7509151

[18] Chen X Liu F Mar Aung Z Zhang Y Chai G. Whole-exome sequencing reveals rare germline mutations in patients with hemifacial microsomia. Front Genet. (2021) 12:580761. doi: 10.3389/fgene.2021.580761, PMID: 34079577 PMC8165440

[19] Chatzakis C Papavasiliou D Mansukhani T Nicolaides KH Charakida M. Maternal vascular-placental axis in the third trimester in women with gestational diabetes mellitus, hypertensive disorders, and unaffected pregnancies. Am J Obstet Gynecol. (2024) 232:S0002937824009001. doi: 10.1016/j.ajog.2024.08.045 39218286

[20] Naeh A Maor-Sagie E Hallak M Toledano Y Gabbay-Benziv R. Greater risk of type 2 diabetes progression in multifetal gestations with gestational diabetes: the impact of obesity. Am J Obstet Gynecol. (2024) 231:259.e1–259.e10. doi: 10.1016/j.ajog.2023.11.1246, PMID: 38360449

[21] Chen Q Zhao Y Shen G Dai J. Etiology and pathogenesis of hemifacial microsomia. J Dent Res. (2018) 97:1297–305. doi: 10.1177/0022034518795609, PMID: 30205013

[22] Da Rosa EB Correia JD Silveira DB Nunes MR Gresele M Dallagnol ME. Risk factors and characteristics of the birth of patients with craniofacial microsomia, a case–control study. Birth Defects Res. (2024) 116:e2289. doi: 10.1002/bdr2.2289, PMID: 38126133

[23] Wood C. Combining evidence-based interventions to support mental health in neonatal intensive care families. Neonatal Netw. (2022) 41:325–32. doi: 10.1891/NN-2021-0046, PMID: 36446438

[24] Tilahun BD. Parental stress and associated factors among parents of preterm neonates admitted at neonatal intensive care unit among selected governmental hospitals Addis Ababa, Ethiopia, 2022. An institution-based cross-sectional study. Front Psych. (2024) 15:1377180. doi: 10.3389/fpsyt.2024.1377180, PMID: 39267695 PMC11391205

[25] Smith M. (2023) Informal caregiving: measuring the cost and reducing the burden. Available online at: https://www.soa.org/498ea3/globalassets/assets/files/resources/research-report/2023/informal-caregiving-reducing-burden.pdf (Accessed June 1, 2025).

[26] Černe T Kragelj LZ Turk E Pavlič DR. Experiences of quality of life and access to health services among rare disease caregivers: a scoping review. Orphanet J Rare Dis. (2024) 19:319. doi: 10.1186/s13023-024-03327-2, PMID: 39217366 PMC11365242

[27] Longacre ML Weber-Raley L Kent EE. Cancer caregiving while employed: caregiving roles, employment adjustments, employer assistance, and preferences for support. J Cancer Educ. (2021) 36:920–32. doi: 10.1007/s13187-019-01674-4, PMID: 31858439

[28] Stock NM Costa B Parnell J Johns AL Crerand CE Billaud Feragen K. A conceptual thematic framework of psychological adjustment in caregivers of children with craniofacial microsomia. Cleft Palate Craniofac J. (2024) 62:10556656241245284. doi: 10.1177/10556656241245284, PMID: 38584503 PMC11458819

[29] Bai Y Li Q Cheng KK Caine ED Tong Y Wu X. Prevalence of postpartum depression based on diagnostic interviews: a systematic review and meta-analysis. Depress Anxiety. (2023) 2023:8403222. doi: 10.1155/2023/8403222, PMID: 40224605 PMC11921862

[30] Chappell M Parikh S Reynolds E. Understanding the impact of pediatric single large-scale mtDNA deletion syndromes on caregivers: burdens and challenges. JIMD Rep. (2023) 64:375–86. doi: 10.1002/jmd2.12385, PMID: 37701326 PMC10494495

[31] Shogenji M Yoshida M Kakuchi T Hirako K. Physical, emotional, and financial burdens of toileting assistance for family caregivers in home care settings and factors associated with each burden: a cross-sectional study. Jpn J Nurs Sci. (2024) 21:e12615. doi: 10.1111/jjns.12615, PMID: 39138022

[32] Kuang Y Wang M Yu NX Jia S Guan T Zhang X. Family resilience of patients requiring long-term care: a meta-synthesis of qualitative studies. J Clin Nurs. (2023) 32:4159–75. doi: 10.1111/jocn.16500, PMID: 36030397

[33] Serchuk MD Corrigan PW Reed S Ohan JL. Vicarious stigma and self-stigma experienced by parents of children with mental health and/or neurodevelopmental disorders. Community Ment Health J. (2021) 57:1537–46. doi: 10.1007/s10597-021-00774-0, PMID: 33475886 PMC8531051

[34] Joyce C Ramsammy C Galvin L Leshabane G Liberty A Otwombe K. Experiences of south african caregivers disclosing to their children living with HIV: qualitative investigations. PLoS One. (2022) 17:e0277202. doi: 10.1371/journal.pone.0277202, PMID: 36445899 PMC9707749

[35] Ashtari S Taylor A. Patients with rare diseases and the power of online support groups: implications for the medical community. JMIR Form Res. (2023) 7:e41610. doi: 10.2196/41610, PMID: 37707878 PMC10540027

[36] Supian A Shafie AA Ngu L-H Ayob H Chaiyakunapruk N. Perceptions of patients and caregivers toward the management of rare disease in Malaysia: a qualitative research study. Int J Technol Assess Health Care. (2024) 40:e34. doi: 10.1017/S0266462324000333, PMID: 39444288 PMC11569895

[37] Thombs BD Levis B Carrier M-E Dyas L Nordlund J Tao L. Effects of a support group leader education program jointly developed by health professionals and patients on peer leader self-efficacy among leaders of scleroderma support groups: a two-arm parallel partially nested randomised controlled trial. Orphanet J Rare Dis. (2022) 17:396. doi: 10.1186/s13023-022-02552-x, PMID: 36307891 PMC9616616

[38] Dumbuya JS Zeng C Deng L Li Y Chen X Ahmad B. The impact of rare diseases on the quality of life in paediatric patients: current status. Front Public Health. (2025) 13:1583. doi: 10.3389/fpubh.2025.1531583, PMID: 40196857 PMC11973084

[39] Huh J Park J-S Sodnom-Ish B Yang HJ. Growth characteristics and classification systems of hemifacial microsomia: a literature review. Maxillofac Plast Reconstr Surg. (2024) 46:18. doi: 10.1186/s40902-024-00427-8, PMID: 38733452 PMC11088588

[40] Cano-Rosás M Benito-Cano J Benito-Cano J Diosdado-Cano JM Benito-Duque P Curto A. Multidisciplinary treatment of hemifacial microsomia: several clinical cases. Clin Pract. (2024) 14:2410–8. doi: 10.3390/clinpract14060188, PMID: 39585016 PMC11587098

[41] Schaaf H Kastellis G Hesse G. “Unmet medical needs” in der HNO-heilkunde. HNO. (2025) 73:189–95. doi: 10.1007/s00106-024-01529-5, PMID: 39503849

[42] Pavić J Krznar M Čukljek S Sedić B Ozimec Vulinec Š Kovačević I. The association between healthcare satisfaction and social support and stress, depression, and life satisfaction in female caregivers: the moderating role of dependence of a sick child. Int J Environ Res Public Health. (2024) 21:1245. doi: 10.3390/ijerph21091245, PMID: 39338128 PMC11431563

[43] Birgfeld C Heike C. Craniofacial microsomia. Clin Plast Surg. (2019) 46:207–21. doi: 10.1016/j.cps.2018.12.001, PMID: 30851752

[44] Patil A Chawathey S Malim A. Adequacy of informed consent in elective surgical procedures: a study in a Navi Mumbai tertiary care Centre. Cureus. (2023) 15:e41777. doi: 10.7759/cureus.41777, PMID: 37449289 PMC10337701

